# Risk assessment of aflatoxin B_1_ in herbal medicines and plant food supplements marketed in Malaysia using margin of exposure and RISK21 approaches

**DOI:** 10.1186/s41021-023-00286-1

**Published:** 2023-11-23

**Authors:** Siti Soleha Ab Dullah, Mohd Redzwan Sabran, Ab Hamid Hasiah, Rozaini Abdullah

**Affiliations:** 1 Department of Environmental and Occupational Health, Faculty of Medicine and Health Sciences, 43400 UPM Serdang, Selangor Darul Ehsan Malaysia; 2 Biomedical Research Policy and Strategic Planning Unit, Institute for Medical Research, National Institute of Health, Persiaran Setia Murni, Setia Alam, 40170 Shah Alam, Selangor Darul Ehsan Malaysia; 3 Department of Nutrition, Faculty of Medicine and Health Sciences, 43400 UPM Serdang, Selangor Darul Ehsan Malaysia; 4 Department of Biomedical Sciences, Faculty of Medicine and Health Sciences, 43400 UPM Serdang, Selangor Darul Ehsan Malaysia; 5 Natural Medicines and Products Research Laboratory, Institute of Bioscience, Universiti Putra Malaysia, 43400 UPM Serdang, Selangor Malaysia

**Keywords:** Risk assessment, Aflatoxin B_1_, Herbal medicine, Plant-food supplement, Margin of exposure, RISK21

## Abstract

Aflatoxin B_1_ (AFB_1_) is a mycotoxin produced by several species of *Aspergillus* fungi which can cause liver cancer in animals and humans. This study aims to perform the risk assessment of AFB_1_ in herbal medicines and plant food supplements (PFS) in Malaysian market. A total of 31 herbal medicines and PFS were purchased through online platforms and over the counter using a targeted sampling strategy. Of 31 samples analysed using the ELISA method, 25 (80.6%) were contaminated with AFB_1_ at levels ranged from 0.275 to 13.941 μg/kg. The Benchmark Dose Lower Confidence level of 10 (BMDL_10_) of 63.46 ng/kg bw/day and the estimated dietary intake of the adult population ranged from 0.006 to 10.456 ng/kg bw/day were used to calculate the Margin of Exposure (MOE). The MOEs for 24 (96%) out of the 25 positive samples were lower than 10,000. The RISK21 matrix revealed that AFB_1_ exposure levels from herbal medicines and PFS differed greatly over the world. The calculated population risk of acquiring liver cancer from AFB_1_ exposure ranged from 0 to 0.261 cancers/100,000 populations/year and accounted for an estimated percentage of liver cancer incidence ranged from 0.002 to 4.149%. This study revealed a moderate risk of liver cancer attributable to AFB_1_ from herbal medicine and PFS among Malaysian populations and emphasised an urgency for risk management actions.

**Supplementary Information:**

The online version contains supplementary material available at 10.1186/s41021-023-00286-1.

## Highlights

- 80.6% of samples analysed were positive with AFB_1_
- Margin of exposure values below 10,000 for 96% of positive samples indicating a high priority for risk management actions
- The RISK21 framework is a helpful tool for communicating and visualising risk
- The estimated percentage of liver cancer incidence attributable to AFB_1_ through consumption of herbal medicine and plant food supplement (PFS) samples ranged from 0.002 to 4.149% revealed that Malaysians were at moderate risk of developing hepatocellular carcinoma

**Supplementary Information:**

The online version contains supplementary material available at 10.1186/s41021-023-00286-1.

## Introduction

Aflatoxins are hazardous secondary metabolites produced by *Aspergillus* types of fungus known as *A. flavus, A. parasiticus, A. nomius,* and *A. tamarii* [1]*.* The contamination of these fungi can occur in the field, during harvest, storage, and processing which can cause substantial health problems to animals and humans. Among many types of aflatoxins, aflatoxin B_1_ (AFB_1_) is the most potent and has been classified by the International Agency of Research on Cancer as a group 1 carcinogen [2]. The AFB_1_ outbreak in Malaysia occurred in the 1960s where the disease spread out in two pig farms in Malacca due to contamination of animal feed [3]. In 1988, 13 children died from eating AFB_1_-contaminated *Loh Shi Fun* noodles which were served during the Nine Emperor Gods festival in Perak, Malaysia [4].

Aflatoxin exposure may be responsible for roughly 25,200–155,000 of the 550,000–600,000 new hepatocellular carcinoma (HCC) cases diagnosed each year worldwide [5]. Further studies discovered the carcinogenic effects of AFB_1_ which have been attributed mostly to the intermediate metabolite AFB_1_-Exo-8,9 epoxide (AFBO) produced from AFB_1_ metabolism by cytochrome P450 enzymes in the liver [6]. AFBO is an extremely unstable chemical that covalently binds to the DNA forming primary adducts known as AFB_1_ is 8,9-dihydro-8-(N^7^-guanyl)-9-hydroxyaflatoxin B_1_ (AFB_1_-N^7^-Gua) which can further breakdown into a less helix-distorting secondary lesion known as AFB_1_-formamidopyrimidine (AFB_1_-FAPy), that inhibit DNA repair and initiate cancer progressions [7, 8]. Besides, liver cancer can occur in humans as a result of synergistic effects of Hepatitis B virus infection as AFB_1_ could increase the risk of liver cancer up to 30 times higher than in people who are exposed to AFB_1_ or hepatitis B infection alone [9].

Herbal medicines are defined as herbs, herbal materials, herbal preparations and finished herbal products, containing active ingredients parts of plants, or other plant materials, or combinations whereas botanical dietary supplements or often referred to as plant food supplements (PFS) are products made from plants, plant parts, or plant extracts which meant to be consumed and to supplement the diet in several dosage forms, including tablets, capsules, liquids, and powders [10, 11]. Based on a baseline study on the use of traditional and complementary medicine (TCAM) in Malaysia, herbal therapy was most frequently used for the treatment of health problems (88.9%) and to maintain health (87.3%) [12]. In Malaysia, herbal medicine and PFS were registered as medicine or food under the purview of the National Pharmaceutical Regulatory Agency or Food Safety and Quality Division established by Ministry of Health respectively.

A study conducted by Shim et al. [13] in South Korea revealed that herbal medicines were highly contaminated with aflatoxins. It was found that 10 out of 700 samples had total aflatoxins ranged from 12.12 to 108.42 ng/g, which were above the European permissible limit of 10 μg/kg for total aflatoxins. In addition, AFB_1_ contamination in herbal medicines and PFS were also studied from other countries including Thailand [14], China [15], and Brazil [16] had proved that herbal medicines and PFS were highly contaminated with AFB_1_ as some samples had AFB_1_ levels above the permissible regulatory limit of 5 μg/kg and 10 μg/kg for AFB_1_ and total aflatoxins in herbal medicines set by the European Commission Regulation (EC) No 1881/2006 [17].

Moreover, a study on the natural occurrence of AFB_1_ on traditional herbal medicines and PFS, known as “*jamu*” and “*makjun*” from Malaysia and Indonesia reported that 70% of samples were positive with AFB_1_ with an estimated daily intake of 0.022 ng/kg [18]. Although the levels of AFB_1_ were relatively lower than in other countries, the results showed that Malaysian population is still not fully protected against AFB_1_ despite having many regulations applied to the registered products. This study aims to perform a risk assessment of AFB_1_ in Malaysian market and to ensure the safety of herbal medicine and PFS consumed by the community using Margin of Exposure (MOE) approach. We also gathered data from literature, and to better communicate the risk, we used the Risk Assessment in the twenty-first Century (RISK21) matrix to depict the exposure-toxicity data. In addition, liver cancer risk among the population and the percentage of liver cancer cases attributable to AFB_1_ intake in herbal medicine and PFS samples were also determined in this study.

## Materials and methods

Methanol, 99.9% HPLC, LC gradient tested, 4 L was obtained from Fisher Scientific (New Hampshire, United States), RBRP116/100 Aflarhone wide, TS-104-10 Trilogy Dried Standard Aflatoxin B_1_, and RIDASCREEN Aflatoxin B_1_ with 96 wells were purchased from R-Biopharm (Darmstadt, Germany).

### Herbal consumption data and collection of samples for analysis

An extensive literature search on the commonly used herbal medicines and PFS in Malaysia was based on cross-sectional studies from PubMed, Google Scholar, Scopus, and Science Direct, and My journal was carried out using the following keywords: “herbal medicine”, “traditional medicine”, “herb “, and “cross-sectional study”. Samples were collected online or over the counter from September 2019 to February 2020. The samples were selected through targeted sampling based on the database from the cross-sectional studies on the most used herbs in Malaysia. AFB_1_ sampling plan was carried out according to the recommendation from the British Food Standard Agency [19, 20]. The samples were selected based on the commercial availability of the sample, accessibility of the samples, manufacturing year must be within 2019 to 2020, and sample must contain one or a mixture of herbs that were listed as the commonly used herbs in Malaysia. All samples were transported to the Environmental Health Laboratory, Universiti Putra Malaysia, finely ground into powder form, and stored at − 20 °C prior to analysis.

### Methanol extraction and sample clean-up

Five grams of powdered sample was extracted with 25 mL of 70% methanol through centrifugation for 10 min at 4000 rpm. The extract was filtered using Whatman No. 1 filter paper. The filtered solution was carefully mixed with 15 mL distilled water and 0.25 mL of Tween 20. An immunoaffinity column (IAC) was used to filter the entire sample solution (approximately 20 mL). The passed solution was discarded, and the IAC was rinsed with 10 mL of distilled water before gently forcing air through the column using a syringe to remove any remaining fluids. The elution stage was carried out by placing a clean and closable vial right below the column and slowly pouring 1 mL of 100% methanol through it at a rate of 1 drop per second. The filtered sample was diluted with distilled water at a 1:10 ratio. Sample extraction and IAC clean-up were carried out in three independent experiments (*n* = 3) for each type of herbal medicine and PFS. One sample from each of the categories of the tablet, liquid, and herbal medicines was spiked with 10 μg/kg of AFB_1_ standard and replicated using the same extraction and sample cleansing techniques. The percentage of recovery was calculated by dividing the measured concentration of the spiked sample by the spiking concentration, multiplied by 100. The recovery rate from spiked samples was used to assess the extraction efficacy and correction of data.

### Quantification of AFB_1_ contamination level using ELISA

The quantification of AFB_1_ in herbal medicines and PFS was carried out using the Ridascreen AFB_1_ ELISA kit (R-Biopharm, Germany) according to the in-house method by R-Biopharm [21]. Fifty μL of diluted sample or standard was carefully pipetted into the wells, followed by 50 μL of conjugate solution and 50 μL of antibody solution, respectively. The sample, conjugate, and antibody solutions were mixed in a well and incubated at room temperature for 30 min. All solutions in the well were discarded after the incubation period. The plate was tapped three times and rinsed with 250 mL of buffer solution to eliminate all residuals from these solutions. Before the addition of 100 μL substrate solution, the plate was tapped 3 times to ensure the wells were free from any residue. After 15 min of incubation at room temperature with the substrate solution, 100 μL of stop solution was added to the well. The plate was read using an ELISA microplate reader (Tecan Group Ltd., Switzerland) at 450 nm wavelength shortly after the stop solution was added. The quantitative analysis was done in three independent experiments (*n* = 3).

For validation of the ELISA assay, the concentration curves of the known AFB_1_ standards were fitted against 1/absorbance using polynomial regression to generate the calibration curve. The limit of quantification (LOQ) was estimated as 10σ / S and the limit of detection [10] as = 3.3σ / S, where σ is the standard deviation of the response and S is the slope of the calibration curve [22]. The percent recovery of the spiked samples with 10 μg/kg AFB_1_ standard was used to determine the extraction efficiency. The spiked samples were subjected to a similar sample preparation and AFB1 quantification method as the analytical samples. Equation 1 is used to calculate the percent recovery.

Percentage of recovery

1 $${Recovery\;\left(\%\right)}=\frac{Spiked\;sample\;conc.\left(\mu g/kg\right)\;\times\;Unspiked\;sample\;conc.\left(\mu g/kg\right)}{Concentration\;of\;AFB1\;added\;to\;the\;spiked\;sample\;\left(\mu g/kg\right)}\times\;100$$

### Estimation of daily intake of AFB_1_

The dietary exposure of AFB_1_ through consumption of herbal medicine and PFS was calculated by multiplying the AFB_1_ contamination level and the daily dose of a sample divided by the average body weight of Malaysians (Eq. 2). The information on the daily dose of the PFS was obtained from the recommended dose on the product packaging whereas the daily dose for herbal medicine was obtained based on the advice of traditional herbal practitioners or suppliers. The average body weight of Malaysian adults was 62.65 kg [23]. However, a 60 kg average body weight was used to ease the calculation [24].

Estimated daily intake (EDI) of AFB_1_ through herbal medicine and PFS consumption

2 $$EDI=\frac{Contamination\;level\;\left(\mu g/kg\right)\;\times\;Daily\;amount\;consumed\;\left(\mu g/kg.bw/day\right)}{Body\;weight\;\left(kg\right)}$$

### Qualitative and quantitative risk assessment

The present study assessed risk using both qualitative and quantitative methods. Margin of Exposure (MOE) as used as a qualitative approach in this study, whereas the quantitative method included estimating liver cancer risk and determining the percentage of liver cancers caused by AFB1. The MOE of a substance is the ratio of the benchmark dose to its estimated lifetime dietary exposure (eq. 3). In this study, the benchmark dose of 63.46 ng/kg body weight/day was used to calculate MOE [25]. The MOE value above 10,000 indicates a low priority for risk management.

Data from literature studies (Supplementary Data A) included the present study were used to generate the RISK21 plots using RISK21 webtool. Input data were estimate of exposure (μg/kg/day) from the level of AFB_1_ of the positive samples and estimate of toxicity (μg/kg/day) from ranges of points of departure (PODs) of BMDL_10_ values of 0.063 to 5.069 μg/kg bw/day [25–30]. The information on the potency value of 0.3 cancers/100,000 population/year/ng of aflatoxin/kg bw/day for hepatitis B positive individuals (HBsAg^+^) and 0.01 cancers/100,000 population/year/ng of aflatoxin/kg bw/day for hepatitis B negative individuals (HBsAg^−^) as reported by JECFA, Organization [31] and the 5.24% prevalence rate of hepatitis B-positive individuals in Malaysia (HBsAg^+^) as reported by Merican et al. [32] were used to calculate the average potency for adult Malaysia population which resulted in 0.025 cancers/100,000 population/year/ng of aflatoxin/kg bw/day as shown in Eq. 4 [33]. The estimated adult Malaysian population liver cancer risk was calculated based on total dietary exposure of AFB_1_ in herbal medicine and PFS as well as the average population potency (Eq. 5). The percentage of liver cancer attributable to the dietary exposure to AFB_1_ from herbal medicine and PFS was calculated as a ratio of the target population risk to the age-standardised incidence rate for liver cancer of 4.9/100,000 population/year for both sexes [34] as shown in Eq. 6.

Margin of exposure

3 $$\it MOE=\frac{BMDL_{10}\ \left( ng/ kg. bw/ day\right)}{Estimated\ Daily\ Intake\ \left( ng/ kg. bw/ day\right)}$$

Average potency for adult Malaysia population

4 $$\textrm{Average}\ \textrm{target}\ \textrm{population}\ \textrm{potency}:\left(0.3\times 0.0524\ \textrm{HBaAg}+\textrm{prevalence}\ \textrm{rate}\right)+\left(0.01\times 0.9476\ \textrm{HBaAg}-\textrm{prevalence}\ \textrm{rate}\right)=0.025\ \textrm{cancers}/\textrm{100,000}\ \textrm{population}/\textrm{year}/\textrm{ng}\ \textrm{of}\ \textrm{aflatoxin}/\textrm{kg}\ \textrm{bw}/\textrm{day}$$

Target population liver cancer risk

5 $$\text{Target}\;\text{population}\;\text{risk}:\text{Dietary}\;\text{exposure}\;\times\;\text{Average}\;\text{target}\;\text{population}\;\text{potency}\\$$

Percentage of liver cancer attributable to AFB_1_ exposure

6 $${Liver\;Cancer\;\left(\%\right)}=\frac{The\;target\;population\;risk\;per\;year\;per\;\text{100,000}\;population}{Age-standardized\;incidence\;rate\;of\;4.9/\text{100,000}\;population/year}\times100$$

## Results

### Sample for analysis

Based on literature search, the most commonly used herbal plants in Malaysia includes *Panax notoginseng*, *Panax quinquefolius, Astragalus, Allium sativum*, *Zingiber officinale*, *Cotula coronopifolia*, *Oldenlandia diffusa*, *Prunus armeniaca*, *Clinacanthus nutans*, *ophiocordyceps sinensis*, *Ginkgo biloba*, *Makjun, Eurycoma longifolia, Labisia pumila, Croton caudatum, Plumbago zeylanica, Nigella sativa, Tamarindus indica, Curcuma longa, Piper porphyrophyllum, Morinda citrifolia*, *Syzygium polyanthum, Acalypha indica, Alpinia purpurata, Parameria Polyneura*, *Allium cepa, Cymbopogon citratus, Curcuma longa, Lawsonia inermis, Piper betle, Striga asiatica, Orthosiphon aristatus, Centella asiatica*, *Momordica charantia*, *Andrographis paniculata, Azadirachta indica, and Morinda citrifolia* [35–42]. Table 1 summarises the product details of samples in the present study. In total, 19 out of 31 samples were purchased over the counter, such as pharmacies and various retail outlets in the Kuala Lumpur city centre, and another 12 samples were obtained from the online platform. Herbal medicine samples were further categorised into different parts of the plant, such as leaves, fruits, seeds, roots, and bulbs, whereas for PFS, the samples were categorised into different dosage forms, such as capsules, tablets, and liquid.

**Table 1:** Product description of the herbal medicine and PFS analysed in the present study

Code	Dosage form	Direction for use/day	Botanical ingredient/herbs
**Plant Food Supplement**			
T1	Tablet	2 tablets, 2 times/day	*Allium sativum*
T2	Tablet	2 tablets, 2 times/day	*Andrographis paniculata*
T3	Tablet	2 tablet, 3 times/day	*Allium sativum*
T4	Tablet	2 tablets, 1 times/day	*Ginkgo Biloba* Extract
T5	Tablet	2 tablets, 1 time/day	*Allium sativum* powder
T6	Tablet	2 tablets, 2 time/day	*Centella Asiatica* and mixture of Indian herbs
C1	Capsule	2 capsules, 2 times/day	*Centella Asiatica*
C2	Capsule	2 capsules, 2 times/day	*Hippocratea indica, Piper nigrum, Trachyspermum ammi, Quercus infectoria, Labisia pumillia lin*
C3	Capsule	2 capsules, 2 times/ day	*Allium sativum*
C4	Capsule	2 capsules, 3 times/day	*Labisia pumillia, Quercus infectoria, Piper nigrum, Hippocratea indica, Trachyspermum Ammi*
C5	Capsule	2 capsules, 2 times/ day	*Allium sativum, Piper betle, Curcuma longa aeroginosa, Zingiber minus, Cuminum minus*
L1	Liquid	2 spoons 1 time/day	*Momordica charantia, Fructus, Ginkgo biloba, Camellia sinensis*
L2	Liquid	2 spoon 1 times/day	*Ophiocordyceps sinensis*
L3	Liquid	1 spoon 1 time/day	*Ginkgo biloba, Centela asiatica, Vitis vinifera*
L4	Liquid	3 spoons 1 time/day	*Phoenix dactylifera, Nigella sativa*, *Piper betle, Crocus sativus*
L5	Liquid	2 spoons 1 time/day	*Punica granatum, Zingiber officinale, Quercus infectoria. Elephantopus scaber, Plectranthus, Labisia pumila*
**Herbal medicine**			
D1	Leaves	Not Available^a^	*Andrographis paniculata*
D2	Leaves	Not Available^a^	*Orthosiphon aristatus*
D3	Leaves	Not Available^a^	*Azadirachta indica*
D4	Leaves	Not Available^a^	*Morinda citrifolia*
D5	Leaves	Not Available^a^	*Clinacanthus nutans*
F2	Calyx	Not Available^a^	*Hibiscus sabdariffa*
F1	Fruit	Not Available^a^	*Momordica charantia*
F3	Fruit	Not Available^a^	*Helminthostachys zeylanica*
F4	Fruit	Not Available^a^	*Quercus infectoria*
R1	Root	Not Available^a^	*Eurycoma longifolia*
R2	Root	Not Available^a^	*Panax quinquefolius*
R3	Root	Not Available^a^	*Labisia pumila*
S1	Seed	Not Available^a^	*Nigella sativa*
S2	Seed	Not Available^a^	*Trigonella foenum-graecum*
B1	Bulb	Not Available^a^	*Allium sativum*

^a^Daily intake was based on recommendation from the seller

### Level of AFB_1_ in herbal medicine and PFS samples and the resulting EDI

**Fig. 1:**
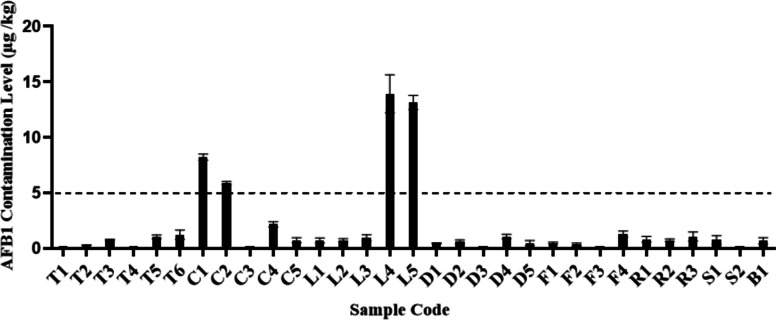
AFB_1_ contamination in herbal medicines and PFS marketed in Malaysia

Figure 1 illustrates the levels of AFB_1_ contamination in herbal medicine and PFS samples obtained from the calibration curve with the function of Y = − 4.5631 × ^3^ + 22.863 × ^2^–10.668x + 1.2652, coefficient correlation of 0.9993, 0.225 μg/kg limit of detection and 0.681 μg/kg limit of quantitation, respectively. The percentage of AFB_1_ recovered from spiked samples was used to evaluate the method’s accuracy. The average recoveries were 90, 91, 82% for tablet, liquid, and crude samples, respectively. Data from the recovery of AFB_1_ were used to calculate the level of AFB_1_ in the collected samples. Of 31 samples analysed, 25 (80.6%, excluding T1, T4, C3, D3, F3, and S2) samples were positive for AFB_1_ at levels ranging from 0.275 to 13.941 μg/kg. Two samples (C1 and C2) from capsule and two samples (L4 and L5) from liquid categories of PFS had AFB_1_ levels ranged from 5.905 to 13.941 μg/kg which exceeded the European regulatory limit of 5 μg/kg. In contrast, all crude herbal medicine samples (D, F, R, S and B) had AFB_1_ levels below the European regulatory limit [17]. The EDI of AFB_1_ from samples were ranged from 0.006 to 10.456 ng/kg bw/day (Table 2).

**Table 2:** AFB_1_ contamination level in herbal medicines and PFS samples and the respective EDI

Sample code	Daily intake (kg or L)	EDI (ng/kg body weight/ day)
**Plant Food Supplement**		
T1	ND ^a^	NA^b^
T2	0.001	0.006
T3	0.003	0.039
T4	ND ^a^	NA^b^
T5	0.002	0.030
T6	0.003	0.064
C1	0.001	0.170
C2	0.001	0.138
C3	ND ^a^	NA^b^
C4	0.003	0.105
C5	0.002	0.020
L1	0.030	0.350
L2	0.030	0.356
L3	0.015	0.232
L4	0.045	10.456
L5	0.030	6.570
**Herbal medicine**		
D1	0.009	0.066
D2	0.001	0.010
D3	ND ^a^	NA ^b^
D4	0.005	0.089
D5	0.005	0.039
F1	0.005	0.039
F2	0.010	0.054
F3	ND ^a^	NA^b^
F4	0.010	0.215
R1	0.002	0.027
R2	0.005	0.061
R3	0.002	0.035
S1	0.020	0.254
S2	ND ^a^	NA^b^
B1	0.009	0.101

^a^Not Detected; ^b^Not available

### Qualitative and quantitative risk assessment of AFB_1_

**Fig. 2:**
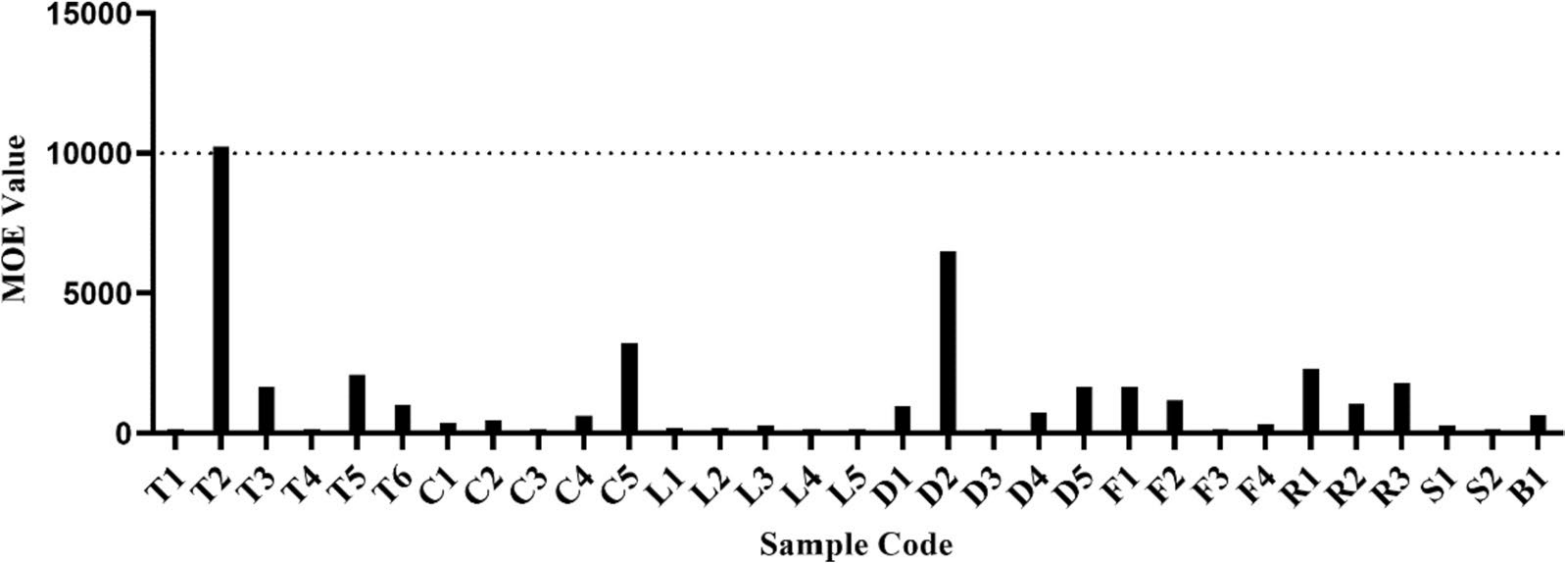
MOE for lifetime exposure to AFB_1_ in herbal medicine and PFS samples

**Fig. 3:**
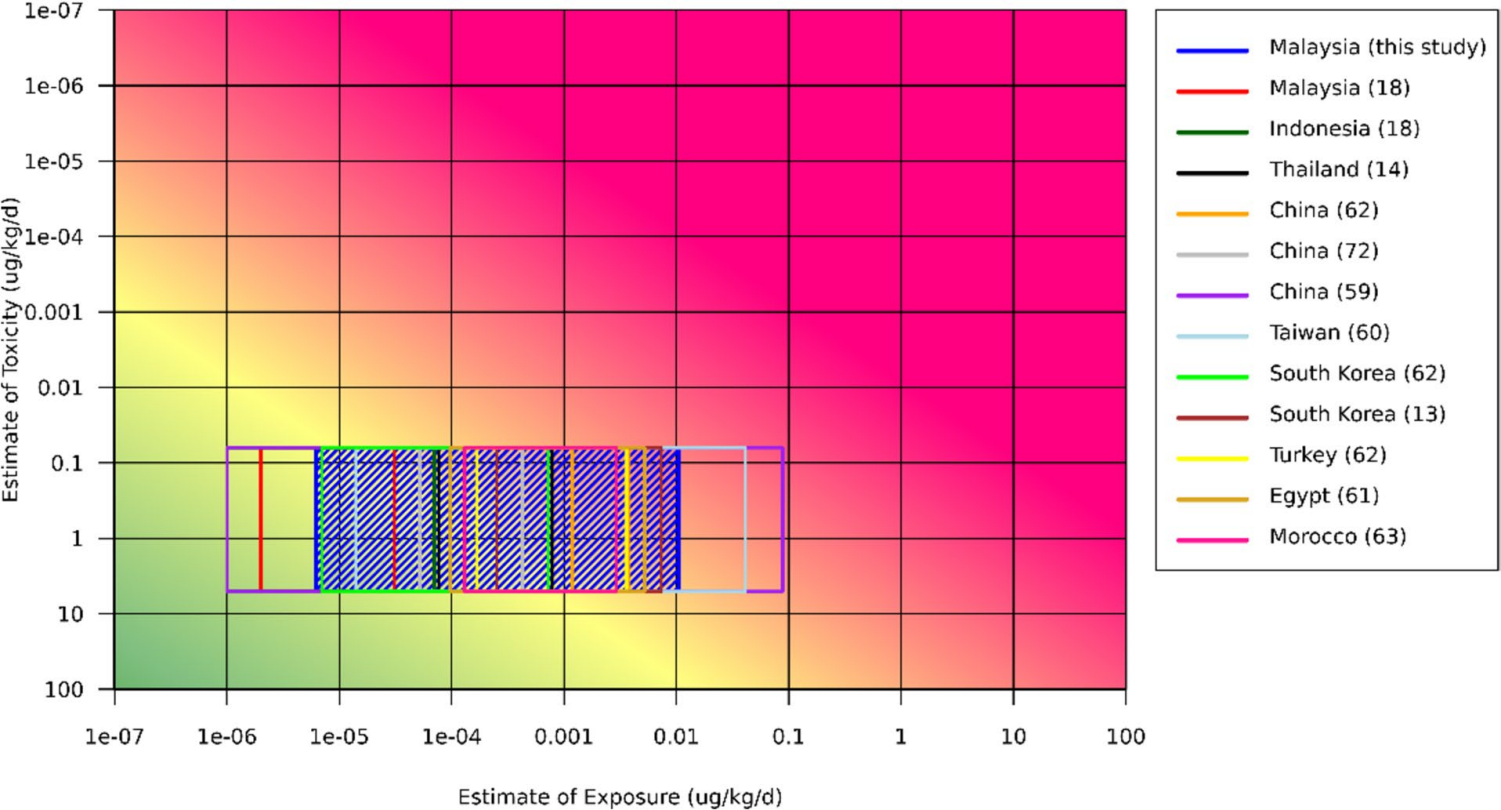
RISK21 plots of estimates exposure of AFB_1_ from different studies and estimates of toxicity from ranges of BMDL_10_ values using RISK21 webtool. The coloured blue box indicates the present study

Figure 2 illustrates the MOEs calculated for lifetime exposure to AFB_1_ that ranged from 6.07 to 10,227.35, with 24 out of the 25 positive samples had MOE less than 10,000. The RISK21 matrix (Fig. 3) was plotted from data from the literature and this study revealed a wide range of AFB_1_ exposure levels from herbal medicines and PFS around the world with different risk levels. The results presented in Fig. 3 reveal a 0.005- to 6.2- fold differences between the range of minimum exposure estimate and 0.003- to 8.4- fold differences between the range of maximum exposure estimate, when comparing with current data. Clearly, more than 50% of positive samples indicated a high priority of risk management actions. Table 3 summarised the estimated liver cancer risk of Malaysians from AFB_1_ exposure through herbal medicine and PFS samples that was 0 to 0.261 cancers/100,000 population/year (upper boundary) as well as the percentage of liver cancer incidence attributable to AFB_1_ exposure from all samples ranged from 0.002 to 4.149%.

**Table 3:** Estimated exposure, cancer risk and percentage cancer incidence attributed to aflatoxins for the general adult population

Exposure (ng/kg bw/ day)^a^ (ng/kg b.w./day)		Estimated cancer risk^b^ (no. of cancers/100,000 population/year)		% Cancer incidence^c^ attributable to AFB1 Exposure	
Lower Boundary	Upper Boundary	Lower Boundary	Upper Boundary	Lower Boundary	Upper Boundary
0.006	10.456	0.000	0.261	0.002	4.149

^a^Based on the mean body weight of the general adult population of 60 kg; ^b^Calculated based on general adult population potency estimate of 0.025 cancers/100,000 population/year per ng/kg b.w./day; ^c^Based on age-standardized incidence rate for liver cancer of 4.9/100,000 population/year [29]

## Discussion

Aflatoxin exposure can lead to life-threatening conditions such as immune system dysfunction, mutagenesis, and cancer [43]. The toxicity and exposure potential of AFB_1_ have been extensively studied [44], and ELISA is the most commonly used method for the detection and quantification of aflatoxin [13, 45, 46]. The ELISA approach is based on the ability of an antibody to recognise the three-dimensional structure of a given aflatoxin. The competitive ELISA approach is often used for the analysis of aflatoxin because this technology is faster and easier to use compared to the HPLC approach [47, 48]. Other advantages of the ELISA assay include high sensitivity and specificity based on antigen-antibody reaction, cost effectiveness, environmental friendliness, and usefulness as a rapid screening method that can analyse a large number of samples in a relatively short time [49, 50].

The present study showed that 80.6% of the samples analysed were contaminated with AFB_1_ at levels ranged from 0.275 to 13.941 μg/kg. Two samples from both capsule and liquid dosage forms of PFS had AFB_1_ levels above the European regulatory limit of 5 μg/kg [17]. According to a study conducted by Shim et al. [13], from 700 herbal medicine samples, 6 samples were contaminated with AFB_1_ above Korea’s regulatory permissible limit of 10 μg/kg for AFB_1_ [51]. The visual inspection of herbal medicine stores reveals the possibility of contamination mainly due to inappropriate storage since there was no freezer or cold room used to store the herbal medicine [13]. Besides, these findings proved that apart from food and spices, herbal medicines and PFS were also susceptible to AFB_1_ contamination. A study conducted in Thailand revealed that AFB_1_ contaminations in all samples of herbal products in various dosage forms were below the National Regulatory Limit of Thailand (20 μg/kg) but some were above the European permissible limit [14]. Moreover, according to the study conducted in Thailand, the highest contamination of AFB1 was found in tablets, which is likely due to contamination of the crude ingredients used to make the tablets [14]. The findings contrasted with the present study since the highest contamination of AFB1 was detected in liquid form of PFS. Nevertheless, it is not easy to determine the relationship between the level of AFB_1_ contamination and the various forms of herbal medicines and PFS marketed in Malaysia and Thailand due to the influence of other environmental factors.

As the use of herbal medicines and PFS are increasing, many countries are facing a significant challenge in monitoring the quality and safety of herbal medicines and PFS sold in the market. According to the World Health Organisation, the lack of quality control and regulation can lead to a high rate of adverse reactions attributable to poor quality herbal medicines, particularly in cases of adulteration with undeclared potent medicinal ingredients and contamination with potentially hazardous contaminants such as AFB_1_ [52, 53]. Besides, many local manufacturers have taken advantage of the interchangeable definitions of herbal products and PFS by registering their products as food to avoid the safety and quality standards required for herbal products. Some products available in the market were not even registered as either herbal products or food, which makes monitoring difficult. We also noticed that the requirements for herbal product registration are focusing on heavy metals content, microbiological contamination, and the use of forbidden herbs. Thus, this study emphasized the need for a specific regulatory limit and requirement standard for AFB_1_ in herbal medicine and PFS especially in Malaysia.

In the present study, risk assessment of AFB_1_ to humans was carried out using dose-response data from animal bioassays, as recommended by the EFSA for establishing the MOE between the BMDL_10_ and human dietary exposure [54]. The benchmark dose analysis requires complete dose-response data to predict the toxicity effects on humans. Although data from human studies is the best way to anticipate the BMDL_10_, purposely exposing humans as a test subject to varying doses of AFB_1_ is unethical. As to support the principle of 3R (reduce, refine and replace the use of laboratory animals), rats’ carcinogenicity data from different studies were used to predict the POD of AFB_1_ using BMDS software resulting in BMDL_10_ values ranged from 63.457 to 5069.239 ng/kg bw/day [25–29]. However, the BMDL_10_ value derived from Wogan et al. [25] was used to calculate the MOE since the study used the most vulnerable sex and species of rats and it produced the lowest BMDL_10_ value for evaluation of the worst-case scenario.

In addition, we also found that the value of BMDL_10_ from Wogan et al. [25] obtained in this study and previous studies by Benford et al. [55], EFSA [56, 57], Gilbert et al. [58], and Leong et al. [24] were different, although similar data set was used. This is due to the daily dose was adjusted using a different method of calculation to compensate for the rat’s standard lifespan (104 weeks). The time-adjusted dose was calculated by multiplying the corrected daily dose by the dosing duration (W) over a period of 104 weeks, according to EFSA [56]. In contrast, European Chemicals Agency considers both the dose duration and the observation time resulted in a lower adjusted dose [59]. Furthermore, the BMD analysis and data interpretation could be different depending on the risk assessor’s judgment, experience, and the EFSA and US EPA’s evolution of BMD analysis guidance. The flexibility in BMR selection and model restriction also can affect the modelling process. Apart from that, the interpretation of the results can be vary based on the best fit criterion. Some criteria can be used to interpret the best-fitting models, including model averaging and a holistic approach that includes not only scientific judgments such as the statistically significant difference of AIC, *p*-value, and BMDL_10_ value but also a visual inspection of the dose-response curve and the BMD: BMDL ratio. Although the BMD: BMDL ratio was not included in either EPA or EFSA guidelines, some researchers used it to highlight the data quality and uncertainty of the dose-response curve in the low-dose zone.

Risk assessment is one of the most effective methods to ensure the safety of herbal medicines and PFS on the market. Dietary exposure was calculated using estimated daily intake (EDI), while risk characterisation was assessed using cancer risk and MOE approach. In the present study, the EDI of AFB_1_ from consumption of herbal medicines and PFS ranged from 0.006 to 10.456 ng/kg bw/day. Compared with the studies conducted in other countries, China had the highest exposure to AFB1 (88.27 ng/kg bw/day) [60], followed by Taiwan (41.19 ng/kg bw/day) [61], South Korea (7.34 ng/kg bw/day) [13], Egypt (5.22 ng/kg bw/day) [62], Turkey (3.59 ng/kg bw/day) [63], Morocco (2.908 ng/kg bw/day) [64], Thailand (0.79 ng/kg bw/day) [14] and Indonesia (0.07 ng/kg bw/day) [18]. It should be noted that AFB_1_ contamination is often unevenly distributed and strongly influenced by environmental factors such as temperature and humidity, as well as climatic changes [65].

In current study, 96% of positive samples had MOEs less than 10,000 indicating a high priority of risk management actions. Margin of exposure is the most appropriate method for characterising risk of carcinogenic and genotoxic substances [57, 66]. The MOE is calculated as benchmark dose derived from a dose-response relationship divided by the estimated intake of a substance. The magnitude of research indicates a level of concern at which a value of 10,000 or higher would be considered a low priority for risk management action, based on the lower benchmark value of 10 (BMDL_10_) from a carcinogenicity study in animals and taking into account many uncertainty factors [56, 57]. The default value of 10,000 was explained by including uncertainty factors of 10 for interspecies, 10 for intraspecies variability in pharmacokinetics and pharmacodynamics, 10 for interindividual uncertainties in cell cycle control and DNA repair, and another factor of 10 for using BMDL_10_ rather than the classical NOAEL [67]. According to EFSA [66], the Scientific Committee stated that “The Scientific Committee is of the view that in general a Margin of Exposure of 10,000 or higher, if it is based on the BMDL_10_ from an animal carcinogenicity study, and taking into account overall uncertainties in the interpretation, would be of low concern from a public health point of view and might be reasonably considered as a low priority for risk management actions…”.

The RISK21 matrix can be a helpful tool for prioritising and communicating risk. This technique offers an adaptable framework for combining knowledge to facilitate effective decision-making [68, 69]. For instance, this framework has been used to prioritise the chemical found in drinking water based on exposure data and toxicity estimates thus provide the valuable additional information for risk assessment [70]. Considering that there are many genotoxic and carcinogenic substances may be exposed via herbal medicine and PFS, therefore, this approach can be used to make informed decisions for human health safety. Another way to analyse the risk of AFB_1_ exposure is by calculating the population risk of liver cancer, which ranged from 0 to 0.261 cancers/100,000 population/year. All samples had an estimated percentage of liver cancer incidence ranging from 0.002 to 4.149% due to AFB_1_ exposure. However, this prediction was lower than the estimated percentage of liver cancer attributable to AFB_1_ calculated by Mohd-Redzwan et al. [71] from the exposure to nuts and nut products of 0.61–14.9% [24], raw peanut of 5.5% [72], and various local foods of 12.4–17.3% [33]. According to the population risk for primary liver cancer and the percentage of liver cancer attributed to AFB_1_ found in this study, Malaysians were at moderate risk of developing primary liver cancer of AFB_1_ exposure through herbal medicine and PFS intake.

In conclusion, AFB_1_ can be found in herbal medicine and PFS on the Malaysian market. The MOE values resulting from consumption of these contaminated samples suggest a high priority for risk management actions especially for long-term exposure to this contaminant.

### Supplementary Information

**Additional file 1.**

## Data Availability

Data and materials presented in the manuscript will be made available upon request to the corresponding author.

## Abbreviations

BMDL_10_: Benchmark dose that gives 10% response
BMD: Benchmark dose
AFB_1_: Aflatoxin B_1_
HCC: Hepatocellular carcinoma
PFS: Plant food supplement
ELISA: Enzyme-linked immunosorbent assay
MOE: Margin of exposure
EDI: Estimated daily intake
AFBO: Aflatoxin B_1_-Exo-8,9 epoxide
IAC: Immunoaffinity Column
BMDS: Benchmark Dose Software
POD: Point of Departure

## References

[1] Benkerroum N. Aflatoxins: producing-molds, structure, health issues and incidence in southeast Asian and sub-Saharan African countries. Int J Environ Res Public Health. 2020;17(4):1215. 10.3390/ijerph17041215 PMC7068566 32070028

[2] IARC (2012). International Agency for Research on Cancer monograph on the evaluation of carcinogenic risks to humans. Chemical agents and related occupations. A review of human carcinogens aflatoxins Lyon.

[3] Hamid AB (1997). Present status and future prospects of research on the groundnut aflatoxin problem in Malaysia.

[4] Cheng CT (1992). Perak, Malaysia, mass poisoning. Tale of the nine emperor gods and rat tail noodles. Am J Forensic Med Pathol.

[5] Liu Y, Wu F (2010). Global burden of aflatoxin-induced hepatocellular carcinoma: a risk assessment. Environ Health Perspect.

[6] Kumar P, Mahato DK, Kamle M, Mohanta TK, Kang SG (2017). Aflatoxins: a global concern for food safety, human health and their management. Front Microbiol.

[7] Chawanthayatham S, Valentine CC, Fedeles BI, Fox EJ, Loeb LA, Levine SS (2017). Mutational spectra of aflatoxin B1 in vivo establish biomarkers of exposure for human hepatocellular carcinoma. Proc Natl Acad Sci U S A.

[8] Geacintov NE, Broyde S (2017). Repair-resistant DNA lesions. Chem Res Toxicol.

[9] Groopman J, Johnson D, Kensler T (2005). Aflatoxin and hepatitis B virus biomarkers: a paradigm for complex environmental exposures and cancer risk. Cancer Biomark.

[10] NTP. National Toxicology Program:Botanical dietary supplements. 2021. Available from: https://ntp.niehs.nih.gov/go/botanical. Accessed 12 May 2022.

[11] WHO (2019). WHO global report on traditional and complementary medicine 2019.

[12] Siti ZM, Tahir A, Farah AI, Fazlin SMA, Sondi S, Azman AH (2009). Use of traditional and complementary medicine in Malaysia: a baseline study. Complement Ther Med.

[13] Shim W-B, Kim K, Ofori JA, Chung Y-C, Chung D-H (2012). Occurrence of aflatoxins in herbal medicine distributed in South Korea. J Food Prot.

[14] Tassaneeyakul W, Razzazi-Fazeli E, Porasuphatana S, Bohm J (2004). Contamination of aflatoxins in herbal medicinal products in Thailand. Mycopathologia.

[15] Liu L, Jin H, Sun L, Ma S, Lin R (2012). Determination of aflatoxins in medicinal herbs by high-performance liquid chromatography–tandem mass spectrometry. Phytochem Anal.

[16] Prado G, Altoé AF, Gomes TC, Leal AS, Morais VA, Oliveira MS (2012). Occurrence of aflatoxin B1 in natural products. Braz J Microbiol.

[17] European Commission (2006). Commission regulation (EC) no. 1881/2006 setting maximum levels for certain contaminants in foodstuffs. J Eur Union.

[18] Ali N, Hashim NH, Saad B, Safan K, Nakajima M, Yoshizawa T (2005). Evaluation of a method to determine the natural occurrence of aflatoxins in commercial traditional herbal medicines from Malaysia and Indonesia. Food Chem Toxicol.

[19] European Commission (2014). Commission regulation (EU) no 519/2014 of 16 may 2014 amending regulation (EC) no 401/2006 as regards methods of sampling of large lots, spices and food supplements, performance criteria for T-2, HT-2 toxin and citrinin and screening methods of analysis. Off J Eur Union.

[20] Food Standard Agency. Mycotoxins sampling guidance. 2016. Available from: https://www.food.gov.uk/sites/default/files/media/document/mycotoxins-sampling-guidance.pdf. Accessed 25 Apr 2022.

[21] R-Biopharm. Enzyme Immunoassay for the quantitative analysis of aflatoxin, B1 Art. No: 1211 and Rida Aflatoxin column Art. No: R5001/5002 [package insert]. Darmstadt: R-Biopharm AG; 1999.

[22] Shrivastava A (2011). Methods for the determination of limit of detection and limit of quantitation of the analytical methods. Chron Young Sci.

[23] Azmi M, Junidah R, Mariam A, Safiah M, Fatimah S, Norimah A (2009). Body mass index (BMI) of adults: findings of the Malaysian adult nutrition survey (MANS). Malays J Nutr.

[24] Leong Y-H, Rosma A, Latiff AA, Ahmad NI (2011). Exposure assessment and risk characterization of aflatoxin B1 in Malaysia. Mycotoxin Res.

[25] Wogan GN, Paglialunga S, Newberne PM (1974). Carcinogenic effects of low dietary levels of aflatoxin B1 in rats. Food Cosmet Toxicol.

[26] Elashoff RM, Fears TR, Schneiderman MA (1987). Statistical analysis of a carcinogen mixture experiment. I. Liver carcinogens. J Natl Cancer Inst.

[27] Wogan GN, Newberne PM (1967). Dose-response characteristics of aflatoxin B1 carcinogenesis in the rat. Cancer Res.

[28] Butler WH, Barnes JM (1968). Carcinogenic action of groundnut meal containing aflatoxin in rats. Food Cosmet Toxicol.

[29] Nixon JE, Sinnhuber RO, Lee DJ, Landers MK, Harr JR (1974). Effect of cyclopropenoid compounds on the carcinogenic activity of diethylnitrosamine and aflatoxin B in rats. J Natl Cancer Inst.

[30] Turley AE, Isaacs KK, Wetmore BA, Karmaus AL, Embry MR, Krishan M (2019). Incorporating new approach methodologies in toxicity testing and exposure assessment for tiered risk assessment using the RISK21 approach: case studies on food contact chemicals. Food Chem Toxicol.

[31] JECFA JFWECoFAM, Organization WH, Food, Nations AOotU, Safety IPoC (1998). Safety evaluation of certain food additives and contaminants / prepared by the forty-ninth meeting of the joint FAO/WHO expert committee on food additives (JEFCA).

[32] Merican I, Guan R, Amarapuka D, Alexander M, Chutaputti A, Chien R (2000). Chronic hepatitis B virus infection in Asian countries. J Gastroenterol Hepatol.

[33] Chin CK, Abdullah A, Sugita-Konishi Y (2012). Dietary intake of aflatoxins in the adult Malaysian population-an assessment of risk. Food Addit Contam Part B.

[34] Ministry of Health M (2006). Malaysian cancer statistics-data and figure, peninsular Malaysia, national Cancer registry.

[35] Shah SA (2016). Prevalence of complementary alternative medicine use among patients with type II diabetes in Negeri Sembilan, Malaysia. Med Health.

[36] Mi LM, Hui C, Zakaria NS, Yusof H (2017). Use of Chinese herbal medicine and health-related quality of life among cancer patients in Johor, Malaysia. Malays J Nutr.

[37] Saw JT, Bahari MB, Ang HH, Lim YH (2007). Herbal use amongst multiethnic medical patients in Penang hospital: pattern and perceptions. Med J Malaysia.

[38] Rahman AA, Sulaiman SA, Ahmad Z, Daud WNW, Hamid AM (2008). Prevalence and pattern of use of herbal medicines during pregnancy in tumpat district, Kelantan. Malays J Med Sci.

[39] Tengku Mohamad TAS, Islahudin F, Jasamai M, Jamal JA (2019). Preference, perception and predictors of herbal medicine use among Malay women in Malaysia. Patient Prefer Adherence.

[40] Kim Sooi L, Lean KS (2013). Herbal medicines: Malaysian women’s knowledge and practice. Evid Based Complement Alternat Med.

[41] Kew Y, Chia YL, Lai SM, Chong KY, Ho XL, Liew DW (2015). Traditional and complementary medicine (TCM) among study population with cardiovascular risk; use and substitution for conventional medicine in Pahang, Malaysia. Med J Malaysia.

[42] Othman C, Farooqui M (2015). Traditional and complementary medicine. Procedia Soc Behav Sci.

[43] Dai Y, Huang K, Zhang B, Zhu L, Xu W (2017). Aflatoxin B1-induced epigenetic alterations: an overview. Food Chem Toxicol.

[44] Hu S, Dou X, Zhang L, Xie Y, Yang S, Yang M (2018). Rapid detection of aflatoxin B_1_ in medicinal materials of radix and rhizome by gold immunochromatographic assay. Toxicon.

[45] Tosun H, Arslan R (2013). Determination of aflatoxin B1 levels in organic spices and herbs. Sci World J.

[46] Mozaffari Nejad AS, Sabouri Ghannad M, Kamkar A (2014). Determination of aflatoxin B1 levels in Iranian and Indian spices by ELISA method. Toxin Rev.

[47] Zheng MZ, Richard JL, Binder J (2006). A review of rapid methods for the analysis of mycotoxins. Mycopathologia.

[48] Horváth E, Pusztahelyi T, Adácsi C, Tanyi E, Pócsi I (2022). Optimization and validation of ELISA for aflatoxin B1 detection in fermented forages and feeds. Scientifica.

[49] Sakamoto S, Putalun W, Vimolmangkang S, Phoolcharoen W, Shoyama Y, Tanaka H (2018). Enzyme-linked immunosorbent assay for the quantitative/qualitative analysis of plant secondary metabolites. J Nat Med.

[50] O’Riordan MJ, Wilkinson MG (2009). Comparison of analytical methods for aflatoxin determination in commercial chilli spice preparations and subsequent development of an improved method. Food Control.

[51] KFDA. Ministry of Food and Drug Safety: Food Code (No.2021–54, 2021.6.29.). 2021. Available from: http://www.mfds.go.kr/. Accessed 13 Mar 2023.

[52] WHO. WHO guidelines on safety monitoring of herbal medicines in pharmacovigilance systems. Geneva: World Health Organization; 2004. Available from: https://apps.who.int/iris/handle/10665/43034. Accessed 16 Mar 2022.

[53] WHO (2007). WHO guidelines for assessing quality of herbal medicines with reference to contaminants and residues.

[54] EFSA. Update: Use of the benchmark dose approach in risk assessment. 2006. Available from: 10.2903/j.efsa.2017.4658. Accessed 2 Apr 2022.

[55] Benford D, Leblanc J-C, Setzer RW (2010). Application of the margin of exposure (MOE) approach to substances in food that are genotoxic and carcinogenic: example: aflatoxin B1 (AFB1). Food Chem Toxicol.

[56] EFSA. Opinion of the scientific panel on contaminants in the food chain [CONTAM] related to the potential increase of consumer health risk by a possible increase of the existing maximum levels for aflatoxins in almonds, hazelnuts and pistachios and derived products 2007 [Available from: 10.2903/j.efsa.2007.446.

[57] EFSA (2020). Risk assessment of aflatoxins in food. EFSA J.

[58] Gilbert Sandoval I, Wesseling S, Rietjens IMCM (2019). Aflatoxin B1 in nixtamalized maize in Mexico; occurrence and accompanying risk assessment. Toxicol Rep.

[59] ECHA. Guidance on information requirements and chemical safety assessment. Chapter R.8: Characterisation of dose [concentration]-response for human health. 2012. Available from: http://echa.europa.eu/web/guest/support/guidance-on-reach-and-implementation. Accessed 29 Apr 2022.

[60] Qin L, Jiang JY, Zhang L, Dou XW, Ouyang Z, Wan L (2020). Occurrence and analysis of mycotoxins in domestic Chinese herbal medicines. Mycology.

[61] Chien M-Y, Yang C-M, Huang C-M, Chen C-H (2018). Investigation of aflatoxins contamination in herbal materia medica in a Taiwan pharmaceutical factory. J Food Drug Anal.

[62] Migahed F, Abdel-Gwad M, Mohamed S (2017). Aflatoxigenic Fungi associated with some medicinal plants. Ann Res Rev Biol.

[63] Lee SD, Yu IS, Jung K, Kim YS (2014). Incidence and level of aflatoxins contamination in medicinal plants in Korea. Mycobiology.

[64] Mannani N, Tabarani A, Abdennebi ELH, Zinedine A (2019). Assessment of aflatoxin levels in herbal green tea available on the Moroccan market. Food Control.

[65] Cotty PJ, Jaime-Garcia R (2007). Influences of climate on aflatoxin producing fungi and aflatoxin contamination. Int J Food Microbiol.

[66] EFSA. Statement on the applicability of the margin of exposure approach for the safety assessment of impurities which are both genotoxic and carcinogenic in substances added to food/feed. EFSA J. 2012;10(3):2578. 10.2903/j.efsa.2012.2578 PMC13151833 42111999

[67] Benford DJ (2015). The use of dose-response data in a margin of exposure approach to carcinogenic risk assessment for genotoxic chemicals in food. Mutagenesis.

[68] Pastoor TP, Bachman AN, Bell DR, Cohen SM, Dellarco M, Dewhurst IC (2014). A 21st century roadmap for human health risk assessment. Crit Rev Toxicol.

[69] Embry MR, Bachman AN, Bell DR, Boobis AR, Cohen SM, Dellarco M (2014). Risk assessment in the 21st century: roadmap and matrix. Crit Rev Toxicol.

[70] Wolf DC, Bachman A, Barrett G, Bellin C, Goodman JI, Jensen E, et al. Illustrative case using the RISK21 roadmap and matrix: prioritization for evaluation of chemicals found in drinking water. Crit Rev Toxicol. 2016;46(1):43–53. 10.3109/10408444.2015.1082973 PMC4732461 26451723

[71] Mohd-Redzwan S, Jamaluddin R, Abd-Mutalib MS, Ahmad Z (2013). A mini review on aflatoxin exposure in Malaysia: past, present and future. Front Microbiol.

[72] Arzandeh S, Selamat J, Lioe H. Aflatoxin in raw peanut kernels marketed in Malaysia. J Food Drug Anal. 2010;18(1): 44–50

